# An Improved Syringe Agroinfiltration Protocol to Enhance Transformation Efficiency by Combinative Use of 5-Azacytidine, Ascorbate Acid and Tween-20

**DOI:** 10.3390/plants6010009

**Published:** 2017-02-14

**Authors:** Huimin Zhao, Zilong Tan, Xuejing Wen, Yucheng Wang

**Affiliations:** 1 State Key Laboratory of Tree Genetics and Breeding, Northeast Forestry University, Harbin 150040, China; huimin0230@163.com; 2 Key Laboratory of Biogeography and Bioresource in Arid Land, Xinjiang Institute of Ecology and Geography, Chinese Academy of Sciences, Urumqi 830011, China; smilebaobao@outlook.com (Z.T.); wxj-329@163.com (X.W.)

**Keywords:** ascorbate acid, 5-azacytidine, syringe agroinfiltration, transformation efficiency, Tween-20

## Abstract

Syringe infiltration is an important transient transformation method that is widely used in many molecular studies. Owing to the wide use of syringe agroinfiltration, it is important and necessary to improve its transformation efficiency. Here, we studied the factors influencing the transformation efficiency of syringe agroinfiltration. The pCAMBIA1301 was transformed into *Nicotiana benthamiana* leaves for investigation. The effects of 5-azacytidine (AzaC), Ascorbate acid (ASC) and Tween-20 on transformation were studied. The β-glucuronidase (*GUS*) expression and GUS activity were respectively measured to determine the transformation efficiency. AzaC, ASC and Tween-20 all significantly affected the transformation efficiency of agroinfiltration, and the optimal concentrations of AzaC, ASC and Tween-20 for the transgene expression were identified. Our results showed that 20 μM AzaC, 0.56 mM ASC and 0.03% (*v*/*v*) Tween-20 is the optimal concentration that could significantly improve the transformation efficiency of agroinfiltration. Furthermore, a combined supplement of 20 μM AzaC, 0.56 mM ASC and 0.03% Tween-20 improves the expression of transgene better than any one factor alone, increasing the transgene expression by more than 6-fold. Thus, an optimized syringe agroinfiltration was developed here, which might be a powerful method in transient transformation analysis.

## 1. Introduction

Genetic transformation is a powerful method used in a variety of molecular studies, such as gene function analysis, protein production, protein–protein interaction and promoter activity. There are two kinds of genetic transformation: stable transformation and transient transformation. Stable transformation is a labor-intensive low-throughput process. Comparatively, transient transformation is an easy and efficient method for gene transformation, which avoids the drawbacks of the stable transformation process, such as transformation efficiency, transformants selection, and regeneration [1]. Additionally, transient expression represents a rapid method to analyze the function of certain genes, and the analysis can be completed within a few days of transformation [2]. Among the transient transformation methods, agroinfiltration is a simple, rapid, versatile and widely used technique. “Syringe infiltration” is the most popular method for agroinfiltration, which is a simple procedure where a needleless syringe is used to introduce *Agrobacterium* into plant leaves, with no need for specialized equipment [3]. 

Syringe infiltration has many advantages, and can either transfer one target gene into the whole leaf, or introduce multiple genes into different areas of one leaf, allowing multiple assays to be conducted on a single leaf [4]. These advantages of syringe infiltration have led to its application in a wide range of studies, such as those concerned with transgenic complementation, promoter analysis, plant pathogen interaction study, abiotic stress tolerance assay, protein production, gene functional analysis with transient overexpressing or silencing assay, protein–protein interactions and protein localization assays [5,6,7,8].

As an important technique, some studies have been performed to improve the transformation efficiency of syringe agroinfiltration. For instance, to defend against viral attack, plant species have developed a post-transcriptional gene silencing (PTGS) system that produces small interfering RNAs to silence non-native genes. Meanwhile, many viruses have developed a system, such as tomato bushy stunt virus (TBSV) p19, which could interfere with the plant [9]. Therefore, *Agrobacterium* cells carrying the p19 protein are frequently co-infiltrated with the *Agrobacterium* carrying the construct of the gene of interest to improve the transgenic transformation efficiency. Dugdale et al. [6] provide a protocol for the design and construction of a split-gene in-plant activation (INPACT). INPACT enables the expression of recombinant proteins at up to 10% of total soluble protein in the leaf within 6 to 9 months. Wroblewski et al. [2] developed a protocol for efficient transient transformation of lettuce, Arabidopsis and tobacco. They found that *Agrobacterium tumefaciens* strain C58C1 did not elicit a necrotic response in plants and was the best strain for these plant species. Similarly, *A. tumefaciens* strain 1D1246 was found to provide high transient expression levels in solanaceous plants without a necrotic response, enabling routine transient expression in solanaceous species. Fujiuchi et al. [10] found that residual water from bacterial suspension in the intercellular space of detached leaves could significantly reduce the yield of recombinant protein expression in the syringe agroinfiltration process, and removal of bacterial suspension water in detached leaves after agroinfiltration significantly improved recombinant protein expression.

Methylation of DNA is found to be involved in the gene expression regulation, and there is an inverse correlation between the methylation level and the transcriptional activity of a gene [11,12]. In addition, DNA methylation level is closely associated with genetic transformation efficiency and transgene expression [12,13,14]. AzaC plays a role in reducing DNA methylation, and could increase the expression of transgenes by decreasing DNA methylation of the transgene [11,15,16]. Therefore, does AzaC also play a positive role in agroinfiltration transformation efficiency? This deserves to be further studied. The genetic transformation of plants mediated by *A. tumefaciens* is a type of pathogenic infection, which normally induces an oxidative burst, with rapid and transient production of reactive oxygen species (ROS) [17]. Excess ROS could be sufficiently toxic to both plant cells and also attack Agrobacterium cells, reducing the efficiency of transformation [17]. Therefore, scavenging ROS is important for genetic transformation, and the antioxidants, such as Ascorbate acid (ASC), may also be involved in enhancing agroinfiltration transformation efficiency. Neutral surfactants, such as Tween-20, Triton X-100 and Silwet L-77, play important roles in reducing surface tension and enhancing the entry of bacteria into plant tissues, and are usually used in genetic transformation, including floral dip transformation or other vacuum infiltration transformation methods [18,19]. However, it is still not known whether it plays a role in enhancing transformation efficiency in syringe agroinfiltration.

Previously, we had built a transient transformation method based on the Agrobacterium mediated method [20,21]. When improving the transformation efficiency of that method, we found that AzaC, ASC and Tween-20 all play roles in enhancing the expression of transgene. Therefore, we supposed that these reagents might also play positive roles in syringe agroinfiltration, and they were further studied in the present study. Our studies showed that 5-azacytidine (AzaC), Ascorbate acid (ASC) and Tween-20 all affect the transformation efficiency of syringe agroinfiltration, and can improve the expression of transgene at a certain level. Furthermore, we found that these factors could coordinately work on genetic transformation. The improvement of syringe infiltration was performed, which will be useful in transient transformation study.

## 2. Results and Discussion

### 2.1. The Effects of AzaC on Transformation Efficiency 

**Figure 1:**
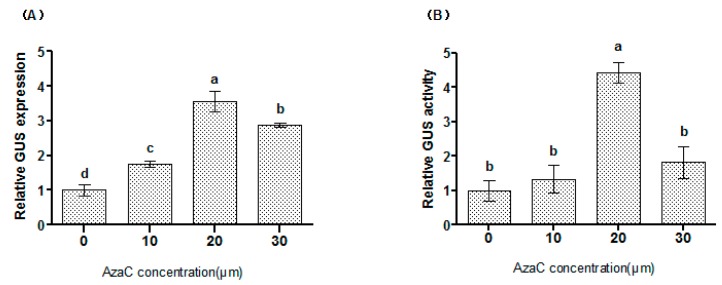
Effects of 5-azacytidine (AzaC) on the transformation efficiency. The expression (**A**) and activity (**B**) of the β-glucuronidase (*GUS*) transgene were analyzed to determine syringe agroinfiltration efficiency. (**A**) *GUS* expression analysis to study the transformation efficiency. The expression of *GUS* in the control (no AzaC supplement) was used as a calibrator to normalize the expression of *GUS* at different concentrations of AzaC (the ratio of the expression of *GUS* at different concentrations of AzaC was divided by the *GUS* expression without AzaC supplement); (**B**) GUS activity analysis to determine the effects of AzaC on syringe agroinfiltration efficiency. GUS activity without AzaC was used as a calibrator to normalize the results of different AzaC levels (the ratio of GUS activity at different concentrations of AzaC was divided by the *GUS* expression without AzaC supplement). a, b, c, d means not sharing a common superscript differ significantly according to ANOVA Tukey’s test (*p* < 0.05).

Different concentrations of AzaC were used for study of the transformation efficiency. Both *GUS* expression and GUS activity measurements showed that AzaC from 10 to 30 μM increased the transformation efficiency, but 20 μM of AzaC increased both the *GUS* expression and GUS activity highest (Figure 1). Therefore, although AzaC at 10–30 μM can affect the expression of transgene, there is an optimal concentration of AzaC for improving the transformation efficiency, and the medium concentration (20 μM) of AzaC improves transformation efficiency more highly than the low (10 μM) or high (30 μM) concentration of AzaC did. 

Previous studies showed that AzaC inhibits and decreases DNA methylation, and treatment with AzaC was found to increase the expression of transgenes by reducing methylation in transferred T-DNA [11,14,15,16]. Additionally, AzaC also inhibits the inactivation of transgene expression. For example, kanamycin-resistant transgenic plants usually lose this resistance over time. However, treatment with AzaC could restore kanamycin-resistance activity and improve the growth of neomycin phosphotransferase-negative plants in the presence of kanamycin [15]. These results indicated that DNA methylation status is quite important in genetic transformation.

Supplement of 20 μM of AzaC significantly increased the transient transformation efficiency (Figure 1), suggesting that AzaC plays an important role in increasing the efficiency of genetic transformation. Previously, Palmgren et al. [12] showed that *Agrobacterium* cells treated with AzaC prior to transformation showed increased transient expression efficiency, which relies on the hypothesis that methylated Agrobacterium DNA will reduce its infection capability, and DNA demethylation could increase T-DNA transformation. Additionally, AzaC could inhibit the methylation-dependent inactivation of the reporter gene in the cells [12]. Therefore, this increased transformation efficiency at 20 μM of AzaC might be the reason that AzaC treatment demethylated the T-DNA, leading to increased transgene expression; and/or AzaC also demethylated Agrobacterium DNA that enhanced its infection capability. Our results also showed that a high level of AzaC decreased the expression of the transgene (Figure 1), perhaps because high AzaC is toxic to plant cells and/or cells of *A. tumefaciens*, which will reduce the transformation efficiency. 

### 2.2. ASC Significantly Affects the Expression of Transgene

**Figure 2:**
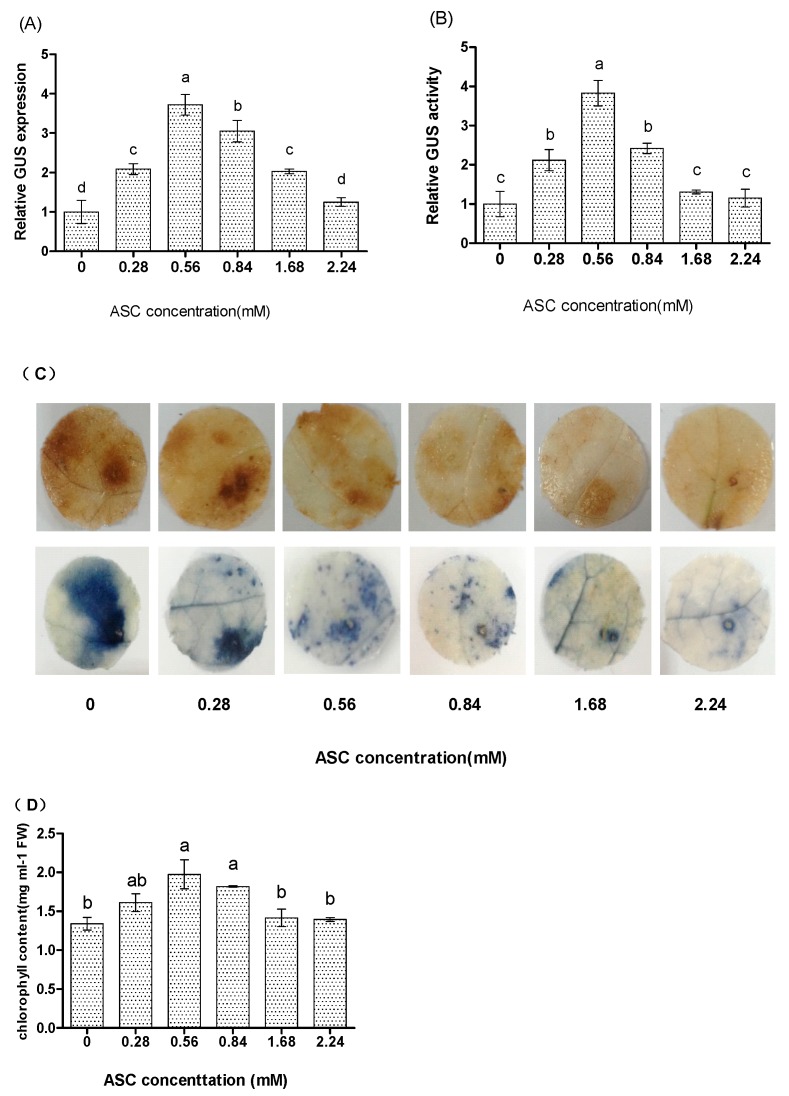
The effects of Ascorbate acid (ASC) on transformation efficiency by monitoring the expression of the *GUS* reporter gene (**A**) and GUS activity (**B**). (**A**) *GUS* expression analysis to study the transformation efficiency. *GUS* expression at different levels of ASC treatment was normalized by that in the control experiment (no ASC was supplied); (**B**) Determination of the effects of ASC on syringe agroinfiltration efficiency by GUS activity analysis. The activity of GUS in the control (without ASC supplement) experiment was used to normalize the activity of GUS at different concentrations of ASC. a, b, c, d means not sharing a common superscript differ significantly according to ANOVA Tukey’s test (*p* < 0.05); (**C**) Analysis of reactive oxygen species (ROS) scavenging by supply of ASC. Detection of ROS by 3-diaminobenzidine (DAB) and nitroblue tetrazolium (NBT) staining that respectively indicate the level of H_2_O_2_ and O^2−^; (**D**) Analyses of chlorophyll contents in the infiltrated leaves supplied with different concentrations of ASC.

GUS activity and expression analysis both indicated that ASC at the concentrations from 0.28 to 1.68 mM all could significantly increase the expression of transgene (Figure 2A), but 2.24 mM of ASC did not affect transformation efficiency (Figure 2A,B). In addition, both *GUS* expression and activity assay showed that there was an optimal concentration for ASC to increase the expression of transgene (Figure 2). The optimum concentration of ASC for increasing the expression of transgene is 0.56 mM, and low or high ASC concentrations were not best for increasing the expression of transgene. These results indicated that the supply of ASC could significantly improve syringe agroinfiltration efficiency at certain concentrations. 

Syringe agroinfiltration in this study is a kind of pathogenic infection in tobacco plants, which will lead to an oxidative burst, with rapid and transient production of reactive oxygen species (ROS) [17]. Excess ROS will cause tissue/cell necrosis, leading to inhibiting regeneration of the transformed cells/tissues, and will inhibit the potential of *Agrobacterium* to colonize plant cells and transfer T-DNA [22,23]. Moreover, the generated ROS could be sufficiently toxic to kill the attacking *Agrobacterium* directly, preventing *Agrobacterium* from transferring T-DNA into plants during attempted transfection [17]. All these will have a negative effect on the transformation efficiency. Therefore, it is important to scavenge the excess ROS during the syringe agroinfiltration process. 

In the present study, we found that the addition of ASC to a certain level could increase the expression and activity of the transformed *GUS* gene significantly during agroinfiltration (Figure 2). For investigating whether ASC increases the transformation efficiency through scavenging ROS, nitroblue tetrazolium (NBT) and 3-diaminobenzidine (DAB) staining were performed. The result showed that O^2−^ and H_2_O_2_ were both reduced, accompanied with the increasing of ASC level (Figure 2C), which reflects the protective effect of ASC against oxidative damage caused by excess ROS accumulation. Abiotic stress will induce the breakdown of chlorophyll, and therefore chlorophyll content can be used as an indicator of plant damage [24]. We further determined the chlorophyll contents in the infiltrated tobacco leaves. The results showed that the infiltrated leaves supplied with 0.28–0.84 mM ASC can retain higher chlorophyll contents than the infiltrated leaves without ASC supplied (Figure 2D). However, the leaves supplied with higher concentrations of ASC (1.68 and 2.24 mM) had similar chlorophyll contents to the leaves without ASC supplied (Figure 2D). This might be the reason why ASC at low and medium concentrations can protect plant cells by reducing excess ROS and is not toxic to plants, resulting in the reduced chlorophyll breakdown. Although a high level of ASC can effectively scavenge ROS, high ASC level is toxic to plant cells, which finally leads to decreased chlorophyll contents. Therefore, these results together might suggest that only a moderate ASC level (0.56 mM) can most highly improve transformation efficiency (Figure 2A,B), which effectively scavenges ROS and is not toxic to plant cells (Figure 2C,D); on the contrary, high ASC level (1.68 mM or more) can effectively reduce ROS accumulation (Figure 2C), but it will be toxic to plant cells, resulting in the breakdown of chlorophyll (Figure 2D), which will cancel out the reduced ROS accumulation, and fail to enhance the transformation efficiency (Figure 2A,B). 

### 2.3. Tween-20 Could Increase the Transformation Efficiency of Syringe Agroinfiltration

**Figure 3:**
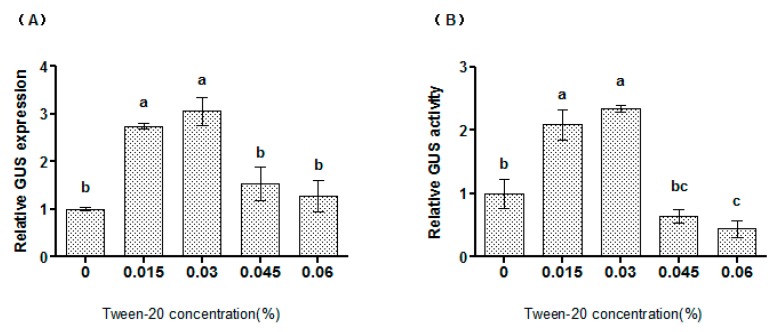
The effects of Tween-20 on syringe agroinfiltration efficiency by determination of *GUS* expression (**A**) and GUS activity (**B**). (**A**) Agroinfiltration efficiency analyzed by *GUS* expression. The expression of *GUS* in the control experiment (no Tween-20 was supplied) was used to normalize the expression of *GUS* at different concentrations of Tween-20; (**B**) The effects of Tween-20 on agroinfiltration efficiency were determined by GUS activity analysis. GUS activity at different levels of Tween-20 treatments was normalized by the level in the control. a, b, c means not sharing a common superscript differ significantly according to ANOVA Tukey’s test (*p* < 0.05).

The influence of Tween-20 on syringe agroinfiltration efficiency was studied. Tween-20 at 0.015% to 0.03% (*v*/*v*) significantly increased *GUS* expression and activity (Figure 3), indicating that Tween-20 affects the efficiency of syringe agroinfiltration. In particular, 0.03% Tween-20 could highly increase the transformation efficiency. However, concentrations of Tween-20 at 0.06% failed to increase the efficiency of syringe agroinfiltration (Figure 3). 

Neutral surfactants such as Tween-20, Triton X-100 and Silwet L-77 are usually used in genetic transformation, because they play important roles in reducing surface tension and enhancing the entry of bacteria into plant tissues [18]. In the present study, the supply of Tween-20 in syringe infiltration improved the transformation efficiency (Figure 3), indicating that Tween-20 can also be used in improving the transformation efficiency. Therefore, Tween-20 in agroinfiltration might increase transformation efficiency through reducing the surface tension, which could enhance the entry of bacteria into plant tissues as shown by the findings of Clough and Bent [18]. However, 0.06% Tween-20 failed to enhance the expression of transgene, and this may be due to the fact that a high level of Tween-20 will damage the plant cells, leading to decreased transformation. 

### 2.4. A Combination of AzaC, ASC and Tween-20 Highly Improves Transformation Efficiency

**Figure 4:**
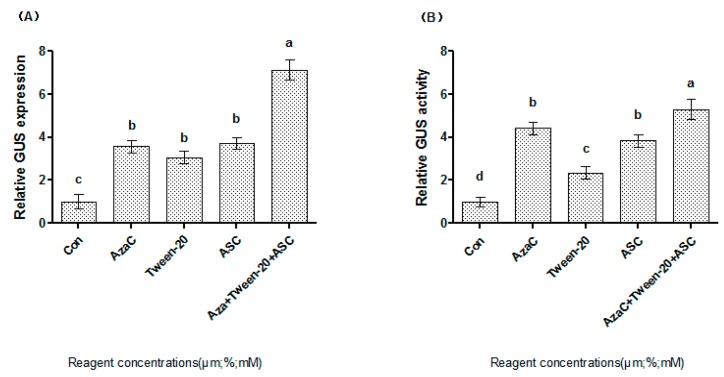
Analysis of the effects of Tween-20, ASC and AzaC together on syringe agroinfiltration efficiency by determination of *GUS* expression (**A**) and GUS activity (**B**). (**A**) Analysis of the effects of 0.03% Tween-20, 20 μM ASC and 0.56 mM AzaC separately or together on syringe agroinfiltration efficiency by *GUS* expression. The expression of *GUS* supplied with Tween-20, ASC and AzaC together was normalized by the expression of *GUS* from the control experiment (no AzaC, ASC and Tween-20 supplied); (**B**) Determination of the effects of AzaC, ASC and Tween-20 together on syringe agroinfiltration efficiency using GUS activity analysis. GUS activity, when supplied with AzaC, ASC and Tween-20 together, was normalized by the activity in the control experiment. a, b, c, d means not sharing a common superscript differ significantly according to ANOVA Tukey’s test (*p* < 0.05).

The above studies showed that AzaC, ASC and Tween-20 could improve syringe agroinfiltration efficiency at certain concentrations (20 μM of AzaC, 0.56 mM of ASC and 0.03% Tween-20). To study whether a combination of these factors could improve the transformation efficiency further, 20 μM AzaC, 0.56 mM ASC and 0.03% Tween-20 were supplied together in the infiltration buffer. Both GUS activity and *GUS* expression analyses showed that these factors together could improve the syringe agroinfiltration efficiency to a greater extent than any of the factors supplied alone (Figure 4), suggesting that AzaC, ASC and Tween-20 could increase the efficiency of syringe agroinfiltration synergistically.

From the above studies, we can see that although the change profiles of *GUS* expression and GUS activities are similar, there were also some differences between them. This should be due to the following reasons. One is that GUS is a very stable enzyme and this might mask some of the differences in gene expression; the other is that GUS activity reflects the post-transcriptional translation of the *GUS* gene that is not the reflection of transcripts of GUS directly. 

## 3. Materials and Methods

### 3.1. Plant Materials and Growth Conditions

The seeds of *N. benthamiana* were planted into the pots containing the mixture of sands and soil (2:1) in a greenhouse under the conditions of 70%–75% relative humidity, 16 h light/8 h darkness photocycle at 25 °C. The 4-week-old plants were used for agroinfiltration. The plasmid pCAMBIA1301 that harbors a *GUS* gene under the control of 35S CaMV promoter was transformed into *Agrobacterium tumefaciens* (EHA105) and was used for the syringe agroinfiltration study.

### 3.2. Infiltration Procedures 

Syringe agroinfiltration was performed according to the method of Broghammer et al. [25] as follows: The *Agrobacterium* strain harboring pCAMBIA1301 was cultured in lysogeny broth (LB) medium containing kanamycin (50 mg/L) and rifampicin (100 mg/L) with rotation at 200 rpm at 28 °C. After the culture reached an optical density at 600 nm (OD_600_) of 0.8, the culture was diluted 50-fold with fresh LB medium, and was cultured at 200 rpm and 28 °C until the OD_600_ reached 0.6. The *Agrobacterium* cells were centrifuged at 3500× *g* for collection, resuspended in infiltration buffer (10 mM MgCl_2_, 10 mM MES, 150 μM acetosyringone, pH = 5.6), adjusted to an OD_600_ of 0.3 and used for syringe agroinfiltration. The mixture (200 μL) was infiltrated into *N. benthamiana* leaves using a needless syringe. The infiltrated regions in leaves were harvested after infiltration for 72 h and used for study. The above procedure was the standard syringe agroinfiltration method, which was used for the following studies. 

### 3.3. Factors Influencing Syringe Infiltration

To improve the transformation efficiency of syringe infiltration, some factors that might affect the transformation efficiency were investigated. We use the infiltration buffer (10 mM MgCl_2_, 10 mM MES, 150 μM acetosyringone, pH = 5.6) as control, and different concentrations of AzaC, ASC and Tween-20 were added in the infiltration buffer for study their effects. To determine the influence of AzaC on syringe agroinfiltration efficiency, 10, 20 and 30 μM of AzaC were added to the infiltration buffer. For determination of the effects of ASC, the stock liquid of ASC (100 mM) was added into the infiltration buffer to reach the concentration at 0.28, 0.56, 0.84, 1.68 and 2.24 mM. To determine the effect of Tween-20 on syringe agroinfiltration efficiency, 0.015, 0.03, 0.045 and 0.06% (*v*/*v*) was supplied in the infiltration buffer. The varying concentrations of these three factors in the infiltration buffer were studied for their effects on agroinfiltration efficiency. To ensure the reliability in experiments, the following measures were performed. To eliminate the physiological difference among the different leaves, the experiment respectively had its control experiment, and the experiment and its control were performed respectively at two sides of the main vein within one leaf. 

To reduce the variations of pCAMBIA1301 amount injected by syringe, the same amount of Agrobacterium cells (i.e., the same OD concentration and same volume, which is shown as Infiltration Procedures) in each experiment had been used. Three independent experiments were performed, and each experiment contains at least five leaves. 

### 3.4. Determination of β-Glucuronidase (GUS) Activity

GUS activity expressed from pCAMBIA1301 was determined according to the method of Jefferson et al. [26]. In brief, leaves were ground into a fine powder in liquid nitrogen and homogenized in extraction buffer (50 mM NaH_2_PO_4_-Na_2_HPO_4_, pH 7.0, 10 mM ethylenediaminetetraacetic acid (EDTA), 10 mM β-mercaptoethanol, 0.1% Triton X-100, 0.1% sodium lauryl sarcosine). Enzyme reactions were performed in extraction buffer supplied with 1 mM 4-methylumbelliferyl-β-d-glucuronide (MUG) at 37 °C and the reaction was stopped by adding 450 μL of 0.2 M Na_2_CO_3_. The fluorescence of 4-methylumbelliferone was monitored using a DyNA Quant fluorometer (Hoefer Pharmacia, San Francisco, CA, USA). A protein standard curve was generated by the Bradford assay.

### 3.5. Quantitative Reverse Transcription PCR (qRT-PCR)

Total RNA was isolated from each sample using the Trizol reagent (Promega), and then treated with DNaseI to remove any DNA contamination. About 1 μg of total RNA was reverse-transcribed into cDNA with oligo deoxythymidine primers using PrimeScript™ RT reagent Kit (Takara, Dalian, China) in a reaction volume of 10 μL, which was subsequently diluted to 100 μL and used as the template for real-time PCR. The *Actin* (GenBank number: AB158612.1) and *α-tubulin* (GenBank number: AB052822.1) genes were used as internal references (see Additional file 1: Table S1 for primers used). The mean Ct of the two internal references was used to normalize the cDNA amount in each sample. Real-time PCR was carried out with an Opticon 2 System (Bio-Rad, Richmond, CA, USA). The reaction mixture contained 10 μL of Synergy Brands (SYBR) Green Real-time PCR Master Mix (Toyobo Co., Ltd., Osaka, Japan), 0.5 μM each of forward and reverse primers, and 2 μL of cDNA template (equivalent to the transcript from 20 ng of total RNA) in a total volume of 20 μL. PCR was performed with the following cycling parameters: 94 °C for 30 s; followed by 45 cycles at 94 °C for 12 s, 60 °C for 30 s, 72 °C for 40 s; and 1 s at 79 °C for plate reading. Melting curves were generated from the samples at the end of each run to assess the purity of the amplified products. Three independent biological replicates were performed, and expression levels were calculated from the cycle threshold according to the 2^−ΔΔ^Ct method [27].

### 3.6. DAB and NBT Staining and Chlorophyll Content Assay

The infiltrated leaves were used for histochemical staining analysis. Infiltration of leaves with 3,3′-diaminobenzidine (DAB) or nitroblue tetrazolium (NBT), which allowed the detection of hydrogen peroxide and superoxide respectively, was performed following the method of Fryer et al. [28]. To measure chlorophyll contents, the infiltrated leaves were ground into fine powder under liquid nitrogen, added with 2 mL of 95% ethanol, incubated in the dark until the material became white, and centrifuged with 12,000 *g* for 2 min. The supernatant was measured at 663 nm and 646 nm, using 95% ethanol as a blank. The concentration (mg/L) of chlorophyll a and b respectively was calculated according to the following formula. Ct: total chlorophyll concentration. Ca = 12.72 D_663_ − 2.59 D_646_, Cb = 22.88 D_646_ − 4.67 D_663_, Ct = Ca + Cb = 20.29 D_646_ + 8.05 D_663_ (Ca and Cb indicate the concentrations of chlorophyll a and b respectively; OD663 and OD645 are the absorbance at wavelength of 663 nm and 646 nm). The Chlorophyll content (mg/g) was calculated as: NCtV/W (where N is the dilution factor; Ct is the total concentration of chlorophyll a and b (mg/L); V is the volume of the extract (mL); W is the fresh weight or dry weight of the sample (g)). 

### 3.7. Statistical Analyses

Statistical analyses were conducted using SPSS 22.0 (SPSS Inc., Chicago, IL, USA) software. Data were compared using ANOVA Tukey’s test (factor analysis of variance). Significant differences from each treatment compared with the control were considered when *p*-value < 0.05.

## 4. Conclusions

As an important and widely use method, it is important to improve the transformation efficiency of syringe agroinfiltration. We studied the factors that significantly influenced the transformation of syringe agroinfiltration. pCAMBIA1301 is used in the transformation study, because the *GUS* gene of pCAMBIA1301 was under the control of the CaMV 35S constitutive promoter that can constantly express the *GUS* gene in different plant tissues at different growth stages; and GUS protein can only be expressed in the eukaryotic cell as introns were present in its sequence. Therefore, the use of pCAMBIA1301 could reflect the transformation efficiency accurately. AzaC, ASC and Tween-20 can all improve the genetic transformation significantly at certain levels. In addition, AzaC, ASC and Tween-20 used together could improve transformation efficiency to a greater extent than any of the three compounds used alone (Figure 4), indicating that these factors could work synergistically to improve the transformation efficiency. Therefore, the optimized conditions for syringe agroinfiltration include 20 μM AzaC, 0.56 mM ASC and 0.03% Tween-20 together, which increases the expression of the transgene by more than 6-fold. 

Due to a RNA-dependent RNA polymerase (RDR1) mutation, *N. benthamiana* is known to be compromised in its ability to silence foreign nucleic acids, which may affect the extrapolation of these results in other species. However, as for the mechanism of these factors in enhancing transformation efficiency and their applications in other genetic transformation methods, AzaC, ASC and Tween-20 could also be used in other plant species. The optimal concentrations may be varied in different plant species, and these need to be optimized. 

## Supplementary Materials

The following are available online at www.mdpi.com/2223-7747/6/1/9/s1, Table S1. The primer sequences used in qRT-PCR.

## References

[1] Guidarelli M., Baraldi E. (2015). Transient transformation meets gene function discovery: The strawberry fruit case. Front. Plant Sci..

[2] Wroblewski T., Tomczak A., Michelmore R. (2005). Optimization of Agrobacterium-mediated transient assays of gene expression in lettuce, tomato and Arabidopsis. Plant Biotech. J..

[3] Santi L., Batchelor L., Huang Z., Hjelm B., Kilbourne J., Arntzen C.J., Chen Q., Mason H.S. (2008). An efficient plant viral expression system generating orally immunogenic Norwalk virus-like particles. Vaccine.

[4] Vaghchhipawala Z., Rojas C.M., Senthil-Kumar M., Mysore K.S. (2011). Agroinoculation and agroinfiltration: Simple tools for complex gene function analyses. Methods Mol. Biol..

[5] Chen Q., Lai H., Hurtado J., Stahnke J., Leuzinger K., Dent M. (2013). Agroinfiltration as an Effective and Scalable Strategy of Gene Delivery for Production of Pharmaceutical Proteins. Adv. Tech. Biol. Med..

[6] Dugdale B., Mortimer C.L., Kato M., James T., Harding R.M., Dale J.L. (2014). Design and construction of an in-plant activation cassette for transgene expression and recombinant protein production in plants. Nat. Protoc..

[7] Yang Y., Li R., Qi M. (2000). In vivo analysis of plant promoters and transcription factors by agroinfiltration of tobacco leaves. Plant J..

[8] Johansen L.K., Carrington J.C. (2001). Silencing on the spot Induction and suppression of RNA silencing in the Agrobacteriummediated transient expression system. Plant Physiol..

[9] Takeda A., Sugiyama K., Nagano H., Mori M., Kaido M., Mise K., Tsuda S., Okuno T. (2002). Identification of a novel RNA silencing suppressor, NSs protein of Tomato spotted wilt virus. FEBS Lett..

[10] Fujiuchi N., Matsuda R., Matoba N., Fujiwara K. (2016). Removal of bacterial suspension water occupying the intercellular space of detached leaves after agroinfiltration improves the yield of recombinant hemagglutinin in a N benthamiana transient gene expression system. Biotechnol. Bioeng..

[11] Bochardt A., Hodal L., Palmgren G., Mattsson O., Okkels F.T. (1992). DNA methylation is involved in maintenance of an unusual expression pattern of an introduced gene. Plant Physiol..

[12] Palmgren G., Mattson O., Okkels F.T. (1993). Treatment of Agrobacterium or leaf disks with 5-azacytidine increases transgene expression in tobacco. Plant Mol. Biol..

[13] Jiang J., Wing V., Xie T., Shi X., Wang Y.P., Sokolov V. (2016). DNA methylation analysis during the optimization of agrobacterium-mediated transformation of soybean. Genetika.

[14] Zhen Z., Karen W.H., Leaf H. (1991). Effects of 5-azacytidine on transformation and gene expression in *Nicotiana tabacum*. Cell. Dev. Biol. Plant.

[15] Christman J.K. (2002). 5-Azacytidine and 5-aza-2′-deoxycytidine as inhibitors of DNA methylation: Mechanistic studies and their implications for cancer therapy. Oncogene.

[16] Weber H., Ziechmann C., Graessmann A. (1990). In vitro DNA methylation inhibits gene expression in transgenic tobacco. EMBO J..

[17] Wojtaszek P. (1997). Oxidative burst: An early plant response to pathogen infection. Biochem. J..

[18] Dan Y. (2008). Biological functions of antioxidants in plant transformation. Cell. Dev. Biol. Plant.

[19] Kuta D.D., Tripathi L. (2005). Agrobacterium-mduced hypersensitive necrotic reaction in plant cells: A resistance response against Agrobacterium-mediated DNA transfer. Afr. J. Biotechnol..

[20] Ji X., Zheng L., Liu Y., Nie X., Liu S., Wang Y. (2014). A Transient Transformation System for the Functional Characterization of Genes Involved in Stress Response. Plant Mol. Biol. Rep..

[21] Zang D., Wang C., Ji X., Wang Y. (2015). *Tamarix hispida* zinc finger protein ThZFP1 participates in salt and osmotic stress tolerance by increasing proline content and SOD and POD activities. Plant Sci..

[22] Clough S.J., Bent A.F. (1998). Floral dip: A simplified method for Agrobacterium-mediated transformation of *Arabidopsis thaliana*. Plant J..

[23] Tague B.W., Mantis J. (2006). In planta Agrobacterium-mediated transformation by vacuum infiltration. Methods Mol. Biol..

[24] Gous P.W., Gilbert R.G., Fox G.P. (2015). Drought-proofing barley (*Hordeum vulgare*) and its impact on grain quality: A review. J. Inst. Brew..

[25] Broghammer A., Krusell L., Blaise M., Sauer J., Sullivan J.T., Maolanon N., Vinther M., Lorentzen A., Madsen E.B., Jensen K.J. (2012). Legume receptors perceive the rhizobial lipochitin oligosaccharide signal molecules by direct binding. Proc. Natl. Acad. Sci. USA.

[26] Jefferson R.A., Kavanagh T.A., Bevan M.W. (1987). GUS fusions: β-glucuronidase as a sensitive and versatile gene fusion marker in higher plants. EMBO J..

[27] Livak K.J., Schmittgen T.D. (2001). Analysis of Relative Gene Expression Data Using Real-Time Quantitative PCR and the 2^−ΔΔCT^ Method. Methods.

[28] Fryer M.J., Oxborough K., Mullineaux P.M., Baker N.R. (2002). Imaging of photo-oxidative stress responses in leaves. J. Exp. Bot..

