# Supplementary material for: An Improved Syringe Agroinfiltration Protocol to Enhance Transformation Efficiency by Combinative Use of 5-Azacytidine, Ascorbate Acid and Tween-20
Source: Plants (Basel). 2017 Feb 14;6(1):9. doi: 10.3390/plants6010009 (PMC5371768; doi:10.3390/plants6010009)
Supplement: Supplementary file 1 [file plants-06-00009-s001.pdf]

Supplementary Materials: An Improved Syringe Agroinfiltration Protocol to Enhance Transformation Efficiency by Combinative Use of 5-Azacytidine, Ascorbate Acid and Tween-20

Huimin Zhao, Zilong Tan, Xuejing Wen and Yucheng Wang

Table S1. The primer sequences used in real-time RT-PCR.

Gene	GenBank Accession Number	Forward Primers (5′-3′)	Reverse Primers (5′-3′)
Actin	AB158612.1	TCCTCATGCAATTCTTCGGT	TTCCAACAAGTGATGGCTGG
α-tubulin	AB052822.1	TCTGAACCGACTTATTTCAC	CATTGACATCCTTTGGCACA
GUS	KM434773	GTCGCGCAAGACTGTAACCA	TGGTTAATCAGGAACTGTTG
